# A New Method of Artificial Respiration

**Published:** 1890-08

**Authors:** 


A NEW METHOD OF ARTIFICIAL RESPIRATION.—Dr. Andrew H. Smith, of New York, writing in the *Virginia Medical Monthly* for April, 1890, describes an improved method of performing artificial respiration. Two persons are required. The patient lying on the back, and, if possible, placed upon a table, one operator at the head takes a hand in each of his and draws the arms directly upward, with a slow and steady pull, continuing the traction until the maximum of thoracic distention is obtained, which will require about three seconds. This accomplished, the traction is relaxed, without, however, attempting to press the arms against the sides. At this moment the other operator, who is kneeling or standing by the side of the patient, presses with both hands forcibly upon the chest in a direction backward and toward the median line, so as to diminish both the depth and the breadth of the lower half of the thorax. This pressure, like the traction, is to be made slowly and steadily, and should be continued until the maximum expiratory result is attained, say two seconds. The pressure is then relaxed, and the traction on the arms follows again immediately. In this way about twelve respiratory movements per minute will be accomplished, under conditions giving the largest excursion of the chest walls attainable by manual procedure. It will be seen that this is a combination of a part of Sylvester's method with the method of Howard. Sylvester's method is defective, in that it does not provide for sufficient compression of the thorax; the position of the operator at the head of the patient making such compression extremely difficult. Howard's plan, on the other hand, does not provide for any expansion of the chest beyond the cadaveric position, and is, upon the whole, much less effective than Sylvester's. A combination of the two, carried out by two person's, secures a very considerable approach to the results of natural respiration, the action of the diaphragm alone being unrepresented. Another very great advantage is the division of labor —either of the other methods being exceedingly fatiguing to the operator.—*Medical Record, June, 1890.*

